# Plerixafor

**Published:** 2012-01-01

**Authors:** Susan Slater

**Affiliations:** From Oregon Health & Science University, Portland, Oregon

Since the early 1980s, high-dose chemotherapy followed by autologous hematopoietic stem cell transplantation (HSCT) has emerged as standard therapy for patients with hematologic malignancies, including non-Hodgkin lymphoma (NHL) and multiple myeloma (MM). In 2009, 32,000 autologous transplants were performed worldwide, 12,000 of which were completed in centers across the United States. Peripheral blood stem cells (PBSCs) were the source of hematopoietic stem cells (HSCs) in 98% of the patients who had undergone transplant (Pasquini & Wang, 2010).

Initially, HSCs were harvested from the recipient’s bone marrow, subjecting the patient to the risks of anesthesia, musculoskeletal discomfort and damage, and blood loss. A shift toward mobilization, or movement, of stem cells from the bone marrow to the peripheral blood and collection of HSCs from the peripheral blood occurred after early studies showed more rapid engraftment after high-dose chemotherapy with peripheral blood stem cell products compared with bone marrow products. As a consequence, the overall period of cytopenia was decreased with concomitant reduction in the need for supportive measures such as blood and platelet transfusions and antibiotic therapy (Bensinger et al., 2001). Over the past decade, mobilization and collection of PBSCs has become standard practice for patients undergoing autologous transplants.

Commonly, stem cells are mobilized from the bone marrow microenvironment to the peripheral blood using either chemotherapy plus high-dose granulocyte colony-stimulating factor (G-CSF; filgrastim [Neupogen]) or high-dose G-CSF alone. US Food and Drug Administration (FDA) guidelines for G-CSF mobilization define a dosage at 10 ìg/kg/day although institutional variations exist. Leukopheresis begins either on recovery of counts postchemotherapy or on day 4 or 5 of G-CSF therapy alone, a time generally associated with the peak migration of HSCs as determined by flow cytometric analysis of the surface expression of the CD34 antigen on peripheral blood mononuclear cells. Daily subcutaneous G-CSF injections and collections continue until the target number of CD34+ HSCs has been collected.

Chemotherapy mobilization results in higher CD34+ cell collections; however, this can be offset by the risk of higher toxicity leading to increased rates of hospitalization for neutropenic fever and infection (Meldgaard Knudsen, Jensen, Gaarsdal, Nikolaisen, & Johnson, 2000). The optimal dose of CD34+ cells remains unclear, but infusion of fewer than 2 × 10^6 ^ CD34+ cells/kg has been associated with delayed engraftment or graft failure, leading to increased morbidity and higher transplant-related costs (Bensinger, DiPersio, & McCarty, 2009). Many factors may influence a patient’s ability to mobilize adequate stem cells, including prior radiation to the marrow space; female gender; premobilization thrombocytopenia; exposure to purine analogs, alkylating agents, or lenalidomide (Revlimid); and marrow involvement by lymphoma (Leis, 2011). Approximately 20% of patients with NHL and MM will fail to collect the minimum CD34+ cell dose required to proceed with transplant (Pusic et al., 2008). Many often require remobilization, accomplished by multiple methods, the most common utilizing the combination of G-CSF plus granulocyte macrophage colony-stimulating factor (sargramostim [Leukine]), with or without concomitant chemotherapy.

## Mechanism of Action

Plerixafor (Mozobil) is a novel small molecule that promotes the mobilization of HSCs. It inhibits the binding of the chemokine receptor CXCR4, which is expressed on HSCs, to its ligand, stromal cell–derived factor-1á (SDF-1á), secreted by bone marrow stroma cells (Cashen, 2009). The binding of SDF-1á to CXCR4 results in the anchoring of stem cells to the bone marrow matrix. Inhibition of this binding results in the release of HSCs into the peripheral blood, where they can then be collected and cryopreserved for later use.

## Indications for Use

Based on two pivotal phase III studies that will be described below, plerixafor was approved by the FDA in December 2008 for use in combination with G-CSF for mobilization of peripheral blood stem cells in patients with NHL and MM (Genzyme Corporation, 2008). Additionally, safety and efficacy have been demonstrated in a phase II study of patients with Hodgkin disease (Cashen et al., 2008). Plerixafor has also been used for HSC mobilization in patients with other diseases such as amyloidosis and germ cell malignancies.

A small pilot study (N = 25) was conducted using plerixafor alone to assess the safety and efficacy of stem cell mobilization in healthy allogeneic sibling donors. Successful collection of sufficient HSCs occurred in two thirds of patients after one apheresis, with the remaining one third achieving sufficient collection after a second apheresis (Devine et al., 2008). Phase II studies using plerixafor alone and in combination with G-CSF in sibling donors for allogeneic HSCT are currently underway through the National Cancer Institute (2011) and the Center for International Blood & Marrow Transplant Research (2010).

It is important to note that plerixafor is not indicated for patients with either acute or chronic leukemia, as its use may cause mobilization of leukemic cells with contamination of the stem cell product (Genzyme Corporation, 2008).

## Clinical Trials

Plerixafor, originally named AMD-3100, was initially investigated as a potential antiviral treatment for patients with HIV/AIDS as the CXCR4 receptor was recognized as the coreceptor for the HIV virus. During phase I trials in healthy volunteers, dosing of plerixafor resulted in a rapid rise in white blood cells expressing the marker CD34, which identified them as HSCs. Additional studies showed a synergistic effect, with plerixafor plus G-CSF resulting in a threefold increase in the numbers of peripheral CD34+ cells compared with G-CSF dosing alone (De Clercq, 2009).

Phase I and II clinical trials were conducted in patients with hematologic malignancies and showed that plerixafor plus G-CSF significantly increased the number of circulating CD34+ cells, resulting in increased CD34+ cell yield from apheresis procedures.

As mentioned previously, two specific phase III trials were critical to the FDA approval of plerixafor in patients with NHL and MM. The first was a multicenter, international trial of 302 patients with multiple myeloma. All participants received G-CSF 10 ìg/kg/day SC daily, then were randomly assigned to receive either plerixafor or placebo beginning on the evening of day 4 and continuing for up to 4 days or until 6 × 10^6 ^ CD34+ cells/kg were collected. A total of 71.6% of the plerixafor-treated patients completed collection in ≤ 2 days, while only 34% of patients in the placebo group were able to complete collection in ≤ 2 days. Over half of the plerixafor-treated patients achieved this goal after one apheresis, while 56% of the placebo-treated patients required 4 apheresis days to meet this goal. Median time to engraftment was similar in both groups, as was 1-year survival (DiPersio et al., 2009a).

The second trial involved 298 patients with NHL and again randomized participants to receive either plerixafor or placebo beginning on the evening of day 4 of G-CSF 10 ìg/kg/day. The target collection was 5 × 10^6 ^ CD34+ cells/kg, with a goal of achieving this target with ≤ 4 apheresis procedures. Again, a significantly larger percentage (87%) of the plerixafor-treated group collected ≥ 2 × 10^6 ^ CD34+ cells/kg in ≤ 4 apheresis procedures, compared with the placebo-treated group (47%). Median time to engraftment and overall survival at 1 year were similar in both groups (DiPersio et al., 2009b).

Of note, both studies offered a "rescue" procedure for those patients who failed to collect either ≥ 0.8 × 10^6 ^ CD34+ cells/kg after 2 days or ≥ 2 × 10^6 ^ CD34+ cells/kg after 4 days. After 7 days of rest, patients were then remobilized with G-CSF 10 ìg/kg/day with plerixafor dosed on the evening of day 4. A full 100% (n = 7) of the MM patients and 60% (n = 62) of the NHL patients who participated in the rescue protocol were able to collect ≥ 2 × 10^6 ^ CD34+ cells/kg in ≤ 4 days.

## Dosage and Administration

G-CSF at a dose of 10 ìg/kg/day is administered by SC injection for four consecutive days. The recommended daily dose of plerixafor is 0.24 mg/kg by SC injection, not to exceed 40 mg/day, dosed on day 4 of G-CSF. As peripheral CD34+ cell counts peak 10 to 14 hours after administration, plerixafor has generally been dosed in the evening prior to beginning stem cell apheresis (Kessans, Gatesman, & Kockler, 2010). G-CSF and plerixafor dosing should continue daily until a sufficient CD34+ cell count has been achieved, with a maximum dosing of 4 consecutive days (Genzyme Corporation, 2008). Plerixafor is supplied in single-use vials containing 1.2 mL of a 20-mg/mL solution. The approximate wholesale cost for each vial is $7,500 (Physicians Desk Reference, 2009).

In patients with normal renal function, approximately 70% of the dose is excreted in the urine within 24 hours of administration. Due to slower excretion in patients with impaired renal function, a dose reduction to 0.16 mg/kg/day (maximum daily dose of 27 mg) is recommended for patients with a creatinine clearance ≤ 50 mL/min to match systemic exposure in patients with normal renal function (MacFarland, Hard, Scarborough, Badel, & Calandra, 2010).

## Adverse Effects

In phase III clinical trials, the most commonly reported side effects associated with plerixafor were gastrointestinal adverse events, mainly diarrhea and nausea, and injection site reactions of erythema and pruritis. Based on World Health Organization criteria, no grade 4 events were reported. Additional adverse reactions are summarized in Table 1 (Brave et al., 2010). This drug has a low potential for significant drug interactions, as it is not metabolized by the CYP system and does not inhibit or induce any CYP isoenzymes (Kessans, Gatesman, & Kockler, 2010).

Plerixafor-mobilized stem cell products contained a higher percentage of T, B, and NK cells when compared with G-CSF mobilized products, which could theoretically influence the incidence and severity of both acute and chronic graft-vs. host disease in allogeneic transplant recipients (Pusic & DiPersio, 2010). Further clinical trials addressing these questions are being pursued.

## Practical Implications

In a retrospective analysis of patients with MM, NHL, and Hodgkin disease undergoing stem cell mobilization with either chemotherapy plus G-CSF or G-CSF plus plerixafor, investigators found there was no significant difference in either the median total CD34+ cells/kg collected or in the number of days required to reach a target of 5 × 106 CD34+ cells/kg. There was a difference, however, in the predictability of initiation of apheresis, with patients receiving plerixafor able to begin apheresis on their target date. Additionally, chemotherapy-mobilized patients often required weekend apheresis procedures, transfusions, and significantly more doses of G-CSF prior to apheresis; 58% required hospital admission for either chemotherapy administration or neutropenic fevers.

Additional analysis demonstrated that the median cost of mobilization and cryopreservation between the two groups was not significantly different (see Table 2). However, the cost to those patients who required more than one dose of plerixafor to collect adequate numbers of CD34+ cells/kg or who required hospitalization for complications of chemotherapy was higher than median costs reported (Shaughnessy et al., 2011). Higher costs were attributed to patients who required more than one dose of plerixafor or hospitalization for complications following chemotherapy administration. 

**Table 1:**
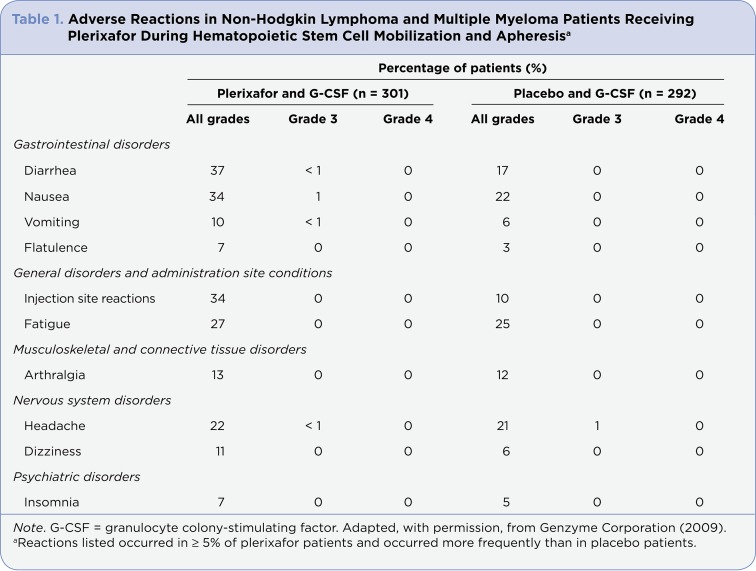
Table 1. Adverse Reactions in Non-Hodgkin Lymphoma and Multiple Myeloma Patients Receiving Plerixafor During Hematopoietic Stem Cell Mobilization and Apheresis

This study was limited by its size (66 patients), its retrospective nature, and limited availability of data evaluating the cost-effectiveness of the use of plerixafor for stem cell mobilization. Additional studies are required to evaluate whether the potential for fewer apheresis days outweighs the higher cost of plerixafor.

## Implications for Advanced Practitioners

Advanced practitioners are frequently responsible for overseeing the mobilization and collection of stem cells in patients preparing for autologous transplant. This includes monitoring peripheral CD34+ counts and initiating apheresis for collection in the appropriate time frame to ensure the best option for adequate collection. The approval of plerixafor for stem cell mobilization provides an additional option to allow more patients to collect stem cells over a shorter period of time, potentially decreasing their overall costs and resulting in fewer failed collections. Advanced practitioners are instrumental in providing patient counseling and nursing staff education, as well as monitoring for side effects and providing supportive care.

## Summary

Plerixafor is a novel agent for use in combination with G-CSF for the mobilization of peripheral blood stem cells in patients with MM and NHL. It has been shown in multicenter randomized trials to decrease the number of apheresis procedures required to achieve a minimum dose of CD34+ cells/kg necessary to proceed with transplant for patients with MM and NHL. Its low side-effect profile makes it well tolerated by a majority of patients with no grade 4 toxicities reported. Future directions include demonstration of safety and efficacy in patients with other malignancies pursuing autologous transplantation and healthy allogeneic donors, as well as additional cost/benefit analysis of the use of plerixafor vs. other mobilization strategies for front-line and rescue mobilization.
